# DPP-4 Inhibitor Sitagliptin Improves Cardiac Function and Glucose Homeostasis and Ameliorates *β*-Cell Dysfunction Together with Reducing S6K1 Activation and IRS-1 and IRS-2 Degradation in Obesity Female Mice

**DOI:** 10.1155/2018/3641516

**Published:** 2018-07-17

**Authors:** Shigang Qiao, Guofang Mao, Hua Li, Zhimin Ma, Lei Hong, Huiling Zhang, Chen Wang, Jianzhong An

**Affiliations:** ^1^Institute of Clinical Medicine Research, The Affiliated Suzhou Hospital of Nanjing Medical University, Suzhou Science and Technology Town Hospital, No. 1 Lijiang Road, Suzhou 215153, China; ^2^Department of Anesthesiology and Perioperative Medicine, The Affiliated Suzhou Hospital of Nanjing Medical University, Suzhou Science and Technology Town Hospital, No. 1 Lijiang Road, Suzhou 215153, China; ^3^Department of Pharmacology, Laboratory of Cerebrovascular Pharmacology, College of Pharmaceutical Science, Soochow University, No. 199 Renai Road, Suzhou 215123, China; ^4^Department of Endocrinology, The Affiliated Suzhou Hospital of Nanjing Medical University, Suzhou Science and Technology Town Hospital, No. 1 Lijiang Road, Suzhou 215153, China

## Abstract

**Background:**

Chronic overnutrition leads to cardiac dysfunction and insulin (INS) resistance. Dipeptidyl peptidase-4 (DPP-4) improves glucose metabolism and insulin sensitivity in both human and animal models. In this study, we explored whether DPP-4 inhibitor sitagliptin (SIT) is involved in the protection of cardiac function and *β*-cell function using an obesity female mouse model.

**Methods:**

Six-week-old C57BL6/J mice were fed a high fat and fructose Western diet with DPP-4 inhibitor SIT for 12 weeks. Cardiac function was examined by echocardiography. Body weight, plasma glucose, and insulin concentrations were measured. The contents of total S6 kinase 1 (S6K1), phosphorylation of S6K1 activation, and INS docking proteins INS receptor substrates 1 and 2 (IRS-1, IRS-2) were assayed, and histology of heart tissue was performed.

**Results:**

Chronic Western diet consumption elevated plasma glucose and insulin and caused obesity, diastolic dysfunction, and *β*-cell dysfunction. DPP-4 inhibition with SIT resulted in reduction in body weight, fasting glucose, and plasma insulin, and improved cardiac diastolic dysfunction. SIT also decreased mTOR/S6K1 activation and prevented the degradation of IRS-1 and IRS-2.

**Conclusions:**

This study revealed pleiotropic protective effects of DPP-4 inhibitor SIT on cardiac function, glycemia, and *β*-cell function together with reducing S6K1 activation and IRS-1 and IRS-2 degradation in the obesity female mouse model.

## 1. Introduction

Overnutrition is defined as excess nutrient supply for normal metabolism and growth. Overnutrition has been linked to both insulin (INS) resistance and *β*-cell dysfunction [1, 2]. The global obesity epidemic is a major reason for the increased incidence of type 2 diabetes mellitus (T2DM) and associated cardiovascular diseases (CVD). Obese individuals may have CVD complications, including hypertension, cardiac diastolic dysfunction, macro- and microvascular disease, and early onset of cardiovascular morbidity [3, 4].

Chronic overnutrition leads to activation of the evolutionarily conserved mammalian target of rapamycin (mTOR) and its downstream signaling molecule, p70 ribosomal S6 kinase 1 (S6K1) [5]. Activation of the INS receptor triggers phosphorylation of tyrosine residues and subsequent activation of the INS docking proteins (INS receptor substrates 1 and 2; IRS-1, IRS-2). It results in the phosphorylation/activation of phosphatidylinositol 3-kinase and protein kinase B (Akt). Tyrosine phosphorylation of IRS-2 appears to play an important role in the antiapoptotic actions of INS in *β*-cells [6]. mTOR/S6K1 signaling impairs INS metabolic signaling through enhanced serine (Ser) phosphorylation of IRS-1, a mechanism implicated in the pathogenesis of INS resistance. Ser phosphorylation of IRS-1 and IRS-2 result in their targeting for proteosomal degradation, which, in turn, leads to impaired INS metabolic signaling [7]. Dipeptidyl peptidase-4 (DPP-4) inactivates and degrades glucagon-like peptide- (GLP-) 1 and glucose-dependent insulinotropic polypeptide (GIP) [8], thereby limiting the favorable metabolic action of these proteins. DPP-4 inhibitors such as sitagliptin (SIT) are a new therapeutic strategy for improving glucose metabolism in patients with T2DM.

SIT inhibits DPP-4, which is responsible for degrading GLP-1. Inhibition of DPP-4 increases circulating levels of GLP-1. GLP-1 is produced by L-cells in small and large intestines in response to nutrient intake [9]. GLP-1 is degraded by DPP-4 in the bloodstream [9]. SIT also regulates GLP-1 receptor and cannabinoid receptor- (CB-) 1 gene expressions, which are associated with appetite regulation in diabetic rat and may decrease oxidative stress and liver tissue damage [10].

Female mice showed that they are more prone to develop obesity than male mice. An 8-week administration of Western diet (WD) abolished the enhancement of insulin sensitivity and induced cardiac diastolic dysfunction in female mice but not in male mice [11]. SIT improves glucose metabolism and insulin sensitivity in both human and animal models [12, 13]. SIT also attenuated the annual exacerbation of diastolic dysfunction in patients with T2DM for 24 months [14]. However, the mechanism underlying this protective effect of SIT remains unclear. In this study, we used the overnutrition obesity female mouse model to evaluate whether a 12-week treatment with SIT improves cardiac diastolic dysfunction and glucose homeostasis and reduces *β*-cell dysfunction. We hypothesized that the SIT may exert its effect through insulin metabolic signaling molecule, p70 ribosomal S6K1, and through phosphorylation of INS docking proteins IRS-1 and IRS-2.

## 2. Methods

### 2.1. Animals

The experimental study was approved by the Suzhou Science and Technology Town Hospital Institutional Animal Care and Use Committee. Six-week-old wild-type control (C57BLKS/J) female mice were purchased from the animal center of Soochow University (Suzhou, China) and were housed under standard laboratory conditions. Groups of 6-week-old mice were fed a WD consisting of high-fat (46%) and a high-carbohydrate component as constituted with sucrose (17.5%) and high-fructose corn syrup (17.5%) and water for 12 weeks with or without SIT (15 mg/kg/day) [15]. Another group of age-matched controls were fed regular mouse chow (CD) with or without SIT for the same period of time.

### 2.2. Biochemical Examination

Venous blood sample was collected from fasting mice for four hours. Glucose and insulin assay were analyzed using an alpha track II glucometer and an ultrasensitive mouse insulin ELISA kit (Crystal Chemical Inc., Wakefield, MA, United States). Samples were drawn immediately prior to the start of the treatment period and at the end of the study. Hemoglobin A1c (HbA1c) was measured using a DCA vantage analyzer (Seimens City, United States). Plasma total cholesterol and triglycerides, serum potassium, and sodium were measured using an Olympus AU680 automated chemistry analyzer (Beckman Coulter, Brea, CA). Triglyceride content in liver samples was also analyzed [16].

### 2.3. Echocardiography

Two-dimensional echocardiograms were performed as described previously [11]. The myocardial performance index (MPI) was calculated as the sum of isovolumic contraction and relaxation times divided by ejection time. The data was obtained by a pulsed-wave Doppler, and parameters were assessed. Calculations were made in accordance with the specific guidelines for rodent echocardiography. All data were acquired and analyzed by a single-blinded observer using Echo PAC (GE Vingmed) offline processing.

### 2.4. INS Signaling Protein Assay

Pancreas islet cells will be isolated by type V collagenase digestion, followed by Ficoll 400 gradient separation, as described in [17]. Proteins involved in INS signaling, including IRS-1, IRS-2, Akt, and S6K1, will be analyzed in pancreatic islets by Western blot.

### 2.5. Hematoxylin and Eosin Staining

Specimens of the hearts were fixed by formalin for 24 hours, dehydrated by 70%, 80%, and 90% ethanol for 3 hours, respectively, then 100% ethanol I for 2 hours and 100% ethanol II for 2 hours, and vitrified by xylene I and xylene II for 20 minutes. After immersing in paraffin I and II for 40 minutes, the specimens were embedded and sliced (5 *μ*m). Staining was performed as follows: hematoxylin staining for 15 minutes, hydrochloric acid alcohol solution for 35 seconds decoloring, eosin staining for 10 minutes, and 90% ethanol for 40 seconds decoloring. Then, neutral balsam was used for mounting, and the section was observed and photographed under the microscope [18].

The heart injuries were divided into three categories. Normal structure: the myocardial structure is normal, the cells are closely aligned, the boundaries are clear, and there is no obvious degeneration, congestion, and edema. Moderate structure injury: the myocardial arrangement was slightly irregular, the myocardial cells were swollen, the myocardial hyperemic edema was visible, and a small amount of lymphocyte infiltration was visible. Serious structure injury: The injury shows loose arrangement of myocardial cell, irregular part of the myocardial fiber fracture, apparent vacuole degeneration, height of myocardial interstitial hyperemia and edema, and a large amount of inflammatory cell infiltration.

### 2.6. Statistical Analysis

All data are expressed as means ± SE. A one-way analysis of variance was used to assess the overall difference between groups. A value of *P* < 0.05 was considered statistically significant.

## 3. Results

### 3.1. Baseline Parameters

In this study, body weight over the course of the 12-week treatment period was elevated in both CD and WD groups. In addition, body weight in the WD group increased more significantly than that in the CD group (*P* < 0.01, Table 1). SIT treatment significantly reduced the elevation of body weight in the WD + SIT group (*P* < 0.05). Body weight and % of body weight gain did not differ between CD and CD + SIT groups after treatment (*P* > 0.05).

### 3.2. Metabolic Parameters

Liver weight, plasma cholesterol, triglycerides, and alanine aminotransferase (ALT) were similarly elevated in the WD group, indicating lipidemia and liver impairment in WD mice (Table 1). Liver weight elevation in WD mice was not reduced by SIT treatment. SIT treatment decreased the elevation of cholesterol, triglycerides, and ALT in WD mice (*P* < 0.05).

### 3.3. Glucose Homeostasis Parameters

**Figure 1:**
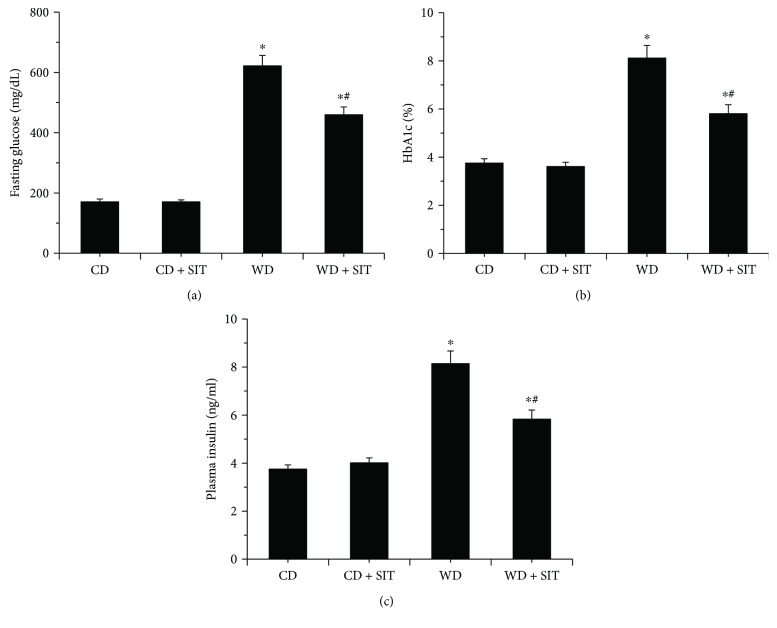
WD mice have elevated fasting glucose and HbA1c levels at the end of treatment. Both fasting glucose (a) and HbA1c (b) were reduced by SIT treatment at the end of the study. WD mice had increased serum insulin concentrations (c). ^∗^*P* < 0.05 compared to CD at the end of experiment; ^#^*P* < 0.05 compared to WD at the end of experiment.

Baseline fasting glucose and HbA1c in all groups did not show significant difference at the beginning of the study. At the end of study, WD groups had higher elevated fasting glucose and HbA1c compared to CD groups (Figures 1(a) and 1(b)), and the fasting glucose and HbA1c in the WD + SIT group were significantly lower compared to those in the WD group (*P* < 0.05). However, the values did not return to the normal range. The results indicate that progressing hyperglycemia was ameliorated by SIT. In this study, mice in the WD group had higher plasma insulin concentrations compared to CD group mice (*P* < 0.01). SIT also reduced the plasma insulin significantly in the WD + SIT group compared to that in the WD group (Figure 1(c)).

### 3.4. WD-Induced Diastolic Dysfunction Was Improved by SIT

**Figure 2:**
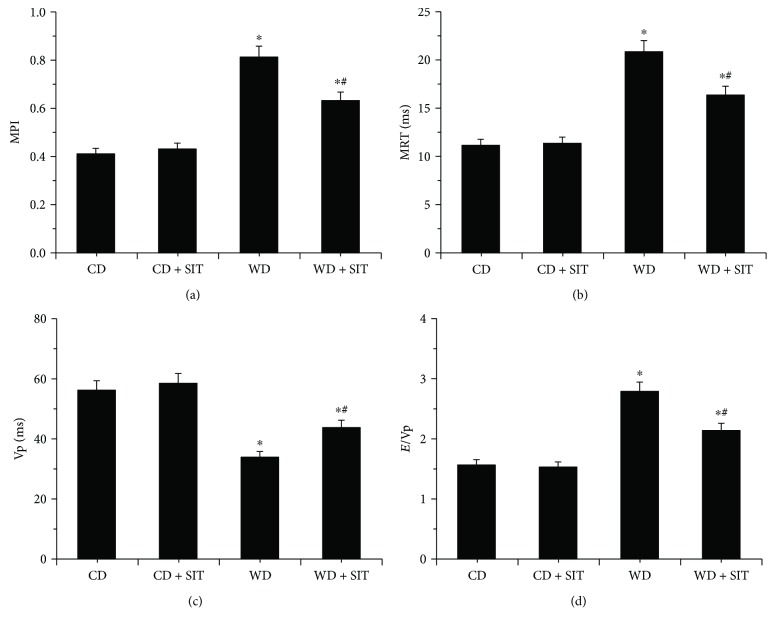
Echocardiographic assessment of cardiac function was evaluated at the end of treatment. WD induces diastolic dysfunction in WD mice. Bar graphs show (a) MPI, (b) isovolumic relaxation time (IVRT), (c) Vp, and (d) *E*/Vp ratio, an index of LV filling pressure. Values are means ± SE. ^∗^*P* < 0.05 compared to CD at the end of experiment; ^#^*P* < 0.05 compared to WD at the end of experiment.

Female mice fed with WD for 12 weeks exhibited abnormal echocardiographic diastolic function parameters when compared with CD and CD + SIT groups (Figures 2(a)–2(d)). The MPI, which assesses both systolic and diastolic function, was increased in the WD group, but its impact was greatly decreased by SIT treatment (Figure 2(a)). An increase in this parameter is indicative of impaired cardiac function. The increase in MPI in the WD group is likely due to abnormal diastolic function as indicated by both a prolonged period of isovolumic relaxation (Figure 2(b)) and a decrease in mitral inflow Vp (Figure 2(c)). The *E*/Vp ratio, a marker of left ventricle filling pressure, was elevated in the WD group compared with CD and CD + SIT groups. This further supports the diagnosis of diastolic dysfunction (Figure 2(d)). SIT treatment effectively decreased this elevation in filling pressure. SIT treatment also significantly ameliorated the impact effects of WD-induced cardiac diastolic function.

### 3.5. WD-Induced Abnormalities in Myocardial Structure Were Improved by SIT

**Figure 3:**
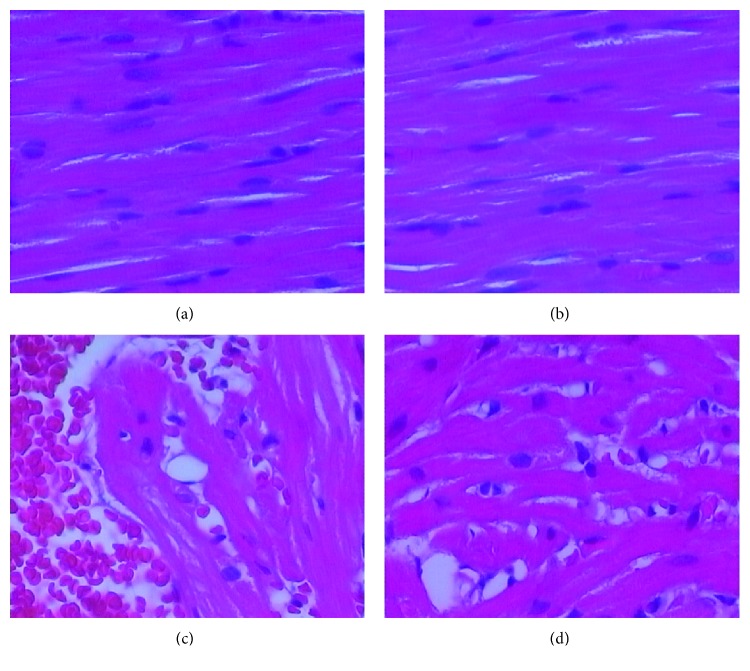
(a) and (b) illustrate the normal appearance of the myocardial structure in the CD group and CD + SIT group. The myocardial structure in WD mice showed a severe myocardial structure injury, including a loose arrangement of myocardial cell, irregular part of the myocardial fiber fracture, apparent vacuole degeneration, myocardial interstitial hyperemia and edema, and a small amount of inflammatory cell infiltration mainly composed of lymphocytes (c). SIT treatment improved the myocardial arrangement, swollen cells, hyperemic edema, and lymphocyte infiltration (d).

The myocardial structure in the CD group and CD + SIT group exhibited normal histology. The cells are closely aligned with clear boundaries. They also do not show degeneration, congestion, and edema (Figures 3(a) and 3(b)). Compared to CD mice, WD mice showed severe myocardial structure injury (Figure 3(c)). In the WD + SIT group, hearts showed a moderate myocardial structure injury. Overall, there is less evidence of cellular injury than in the WD group (Figure 3(d)). The injury of myocardium structure was less severe in WD + SIT group mice than that in WD group mice.

### 3.6. Evaluation of Insulin Signaling in Pancreas

**Figure 4:**
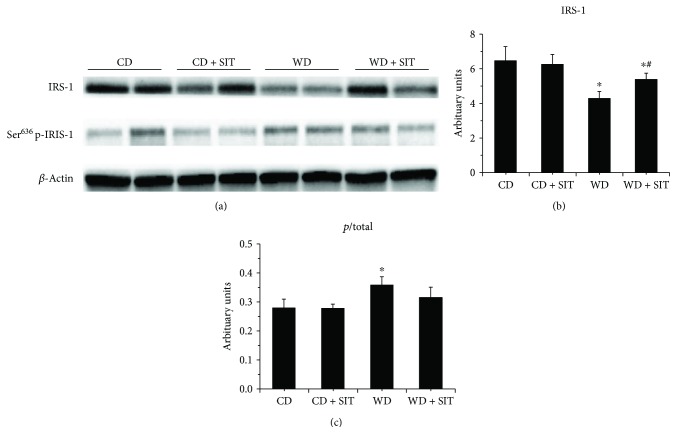
Western blot analysis of phosphorylated (*p*) and total IRS-1 in pancreas tissue at the end of experiment, total IRS-1 decreased, and Ser phosphorylation of IRS-1 increased in WD mice. SIT treatment reduced the total IRS-1 significantly, not the ratio of *p*/total. ^∗^*P* < 0.05 compared to the CD group; ^#^*P* < 0.05 compared to the WD group.

**Figure 5:**
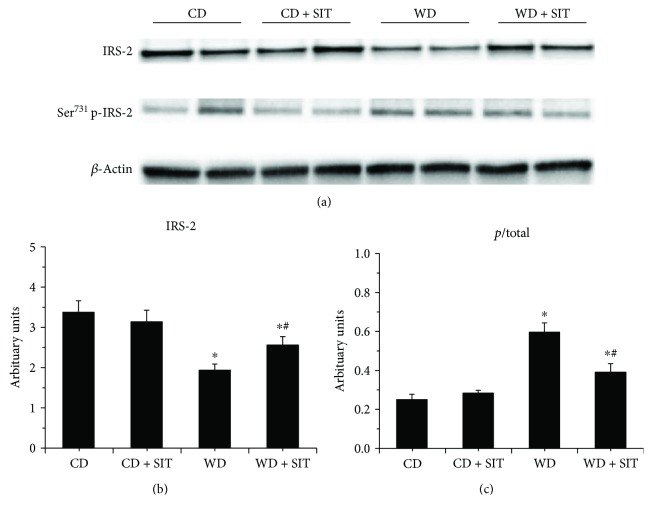
Western blot analysis of phosphorylated (*p*) and total IRS-1 in pancreas tissue at the end of experiment, total IRS-1 decreased, and Ser phosphorylation of IRS-2 increased in WD mice. SIT treatment reduced both the total IRS-2 significantly and the ratio of *p*/total. ^∗^*P* < 0.05 compared to the CD group; ^#^*P* < 0.05 compared to the WD group.

**Figure 6:**
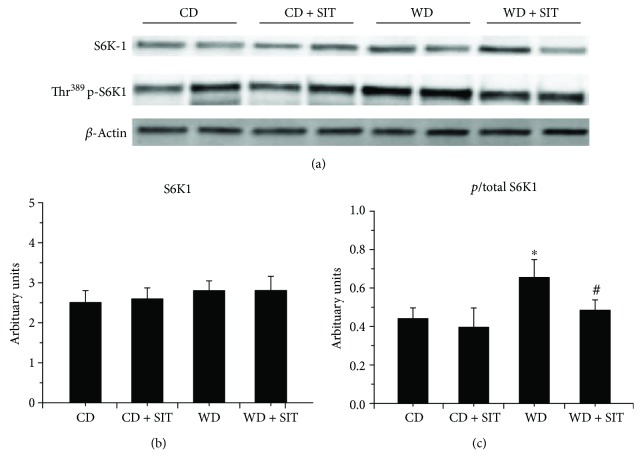
Western blot analysis of phosphorylated (*p*) and total S6K1 in pancreas tissue at the end of experiment. The ratio of thr-phosphorylation of S6K1 over total S6K1 increased in WD mice. SIT treatment reduced the ratio of *p*/total in WD mice significantly. ^∗^*P* < 0.05 compared to the CD group; ^#^*P* < 0.05 compared to the WD group.

In WD fed mice, progressive *β*-cell failure leads to overt hyperglycemia. They usually develop obesity and hyperglycemia due to endocrine pancreatic insufficiency [19]. Western blot evaluation demonstrated decreased total IRS-1 and IRS-2 and an increased phosphorylated Ser^636^ of IRS-1 and Ser^731^ of IRS-2 in pancreas tissue (Figures 4 and 5). SIT treatment reduced the degradation of IRS-1 and IRS-2 (*P* < 0.05) and phosphorylation of IRS-1 and IRS-2. S6K1 phosphorylation was increased in WD fed mice compared to CD counterparts and was decreased by SIT treatment (Figure 6). Our pancreas tissue data confirmed *β*-cell dysfunction in the WD fed mouse and demonstrated excessive activation of S6K1 as well as increased abnormal Ser phosphorylation of IRS-1 and IRS-2, along with degradation of total IRS-1 and IRS-2. Moreover, these alterations were attenuated by SIT treatment.

## 4. Discussion

The aim of the study was to determine whether a 12-week treatment with DPP-4 inhibitor SIT ameliorates overnutrition-induced progression of abnormal cardiac dysfunction and glucose homeostasis in obese female mice. The role of insulin metabolic signaling molecules was also investigated. This study showed that DPP-4 inhibitor SIT improves cardiac diastolic function in female obese mice. This improvement was associated with the protective effect of *β*-cell function by reductions of mTOR/S6K1 activation, degradation, and serphorylation of INS docking proteins IRS-1 and IRS-2. The results show that targeted pharmacologic interventions with DPP-4 inhibitor could be useful in ameliorating pathophysiologic abnormalities in cardiac diastolic dysfunction and preventing the activation of insulin metabolic signaling molecules through enhanced mTOR/S6K1 activation and Ser phosphorylation of IRS-1 and IRS-2. This is one mechanism implicated in the pathogenesis of INS resistance in the setting of obesity or diabetes.

Chronic overnutrition with a WD resulted in obesity, insulin resistance, and elevated plasma DPP-4 activity as well as heart enlargement and dysfunction [20]. Diabetic db/db mice are reported to exhibit increased interstitial fibrosis as early as 2 months of age [21]. We observed an improvement in cardiac diastolic function which was associated with reductions in myocardial fiber fracture, apparent vacuole degeneration, myocardial interstitial hyperemia, edema, and inflammatory cell infiltration in WD fed mice with SIT administration. Cardiac structure in the WD group was more impaired, and this could be due to the result of stress on the left ventricle wall caused by an increase in left ventricle filling pressure. DPP-4 inhibitor linagliptin suppresses reactive oxygen species (ROS) generation, nicotinamide adenine dinucleotide phosphate (NADPH) oxidase, and proinflammatory signals and reduces collagen deposition [22]. There is growing evidence that a DPP-4 inhibitor could exert cardioprotection and improve left ventricular function by reducing oxidative stress and apoptosis and increasing reperfusion injury salvage kinase (RISK) activity [23]. Thus, it is likely that SIT may blunt myocardium injury progression. The underlying mechanism needs to be further investigated.

Multiple metabolic and proliferative pathways implicated in INS resistance lead to Ser phosphorylation of IRS-1. However, the role of the mTOR/S6K1 signaling pathway is particularly interesting because it is affected by nutrients and energy status. Studying this pathway allows for a comprehensive evaluation of the role of overnutrition on INS resistance and *β*-cell failure. Excessive activation of mTOR/S6K1 can also impair INS metabolic signaling through enhanced Ser phosphorylation of IRS-1 and IRS-2, which targets these molecules for proteosomal degradation. This is a widely accepted mechanism which contributes to the pathogenesis of INS resistance in several tissues, including skeletal muscle [24–26]. Our data in the pancreas tissue confirmed *β*-cell dysfunction in the WD fed mouse and demonstrated excessive activation of S6K1 as well as increased abnormal Ser phosphorylation of IRS-1 and IRS-2, along with degradation of IRS-1 and IRS-2. Moreover, these alterations were attenuated by SIT treatment in our obese female mouse model. Other studies have demonstrated increased mTOR/S6K1 activation in the transgenic Zucker obese (ZO) rat [2, 27], a model of overnutrition and obesity which carries a mutation of the leptin receptor and develops INS resistance and glucose intolerance. This suggested that the mTOR/S6K1 and INS metabolic signaling pathways in the pancreas play an important role in the development and survival of *β*-cells. S6K1 KO mice have decreased *β*-cell size and mass, hypoinsulinemia, and glucose intolerance, suggesting a critical participation of this pathway in *β*-cell survival [28]. How DPP-4 inhibitor attenuates the activation of the S6K1 in the setting of overnutrition- and obesity-induced INS resistance as well as its relation to *β*-cell function is yet fully elucidated.

Chronic treatment of WD fed mice with the DPP-4 inhibitor led to marked inhibition of plasma DPP-4 activity and improved insulin sensitivity. DPP-4 inhibition may suppress INS resistance with a decrease in body weight but not back to control levels [20]. In a fatty liver Shionogi-ob/ob male mouse model, SIT administration reduced body weight, blood glucose levels, and hepatic fibrosis. It also attenuated hepatic stellate cell activation and Kupffer cells [29]. Consistent with these studies, we observed the differences in body weight between CD and WD at the end of experiment in our female obesity model. However, in insulin-resistant male ZO rats, DPP-4 inhibitor linagliptin treatment for 8 weeks did not alter the body weight during the study period [30]. This difference in results may due to the different animal strain used (rat versus mouse; male versus female), as well as different inhibitor and treatment time.

In our study, food intake or calorie intake was not measured to evaluate whether beneficial effects of SIT could be due to a decrease in food intake or low-calorie intake. A research study demonstrated that SIT attenuated body adiposity, without affecting food intake, in C57BL/6 mice with diet-induced obesity [31]. However, in a clinical study patients who were given with SIT 100 mg (oral) daily for 4 weeks, postprandial serum glucagon, fasting blood glucose, and 24-h caloric intake decreased [32]. The possibility that SIT exerts its beneficial effects in part via suppression of food intake or low-calorie intake cannot be excluded.

Saxagliptin monotherapy prevented or delayed the progression of impaired glucose tolerance or impaired fasting glucose to type 2 diabetes mellitus in obese patients with newly diagnosed prediabetes [33]. SIT administration decreased ambient blood glucose levels and improved the glucose excursion rate. This was associated with elevated plasma insulin and reduced plasma glucagon levels [34]. SIT also decreased circulating DPP-4 activity, improved glucose tolerance, glucose-stimulated insulin secretion, and insulin sensitivity, and reduced plasma triglycerides and cholesterol levels [35]. In the recent study, SIT protected liver tissue, modulated lipid metabolism in a mouse model, and mediated expression levels of key enzymes for lipid metabolism [15]. Our results also showed that SIT decreased the elevation of cholesterol, triglycerides, and ALT in the WD group. Given that SIT altered the elevation of cholesterol, triglycerides, and ALT in the WD + SIT group compared to the WD group, this indicates that plasma glucose metabolic change may be involved in the improvement effect of SIT on cardiac dysfunction in WD fed female mice. In addition, high-fat fed mice treated with SIT exhibited significant improvement in insulin sensitivity indicating that improvements in glycemic control were not solely a consequence of enhanced insulin secretion [36]. The recent study suggested that the protective effects afforded by this DPP-4 inhibitor may derive from improvement of the metabolic profile and from cytoprotective properties [37]. In the Zucker diabetic fatty (ZDF) rat, Ferreira et al. have found that chronic SIT treatment corrected the glycemic dysmetabolism, hypertriglyceridemia, inflammation, and hypertension and reduced the severity of the histopathological lesions of pancreatic endocrine and exocrine tissues, together with a favorable redox status, which provides a further advantage in the management of diabetes and its proatherogenic comorbidities [38].

In summary, our study supports a newly described pleiotropic protective effect of DPP-4 inhibitor SIT on diastolic function and *β*-cell function in the obesity female mouse model. Despite the improvement in glycemic control, the HbA1c and fasting glucose values remained elevated. It is likely that the pleiotropic effects of SIT relate to factors other than improvements in glycemia and lipidemia. These findings suggest a potential clinical utility for SIT in the obesity/diabetic population. However, some limitations exist in the current study, such as pancreas histomorphology could not be done to study the impact of SIT on pancreas lesions and the indirect effect on dysfunction by assessing S6K1, IRS-1, and IRS-2 was not evaluated. Additional studies are needed to further elucidate the potential role of mTOR/SGK1 and INS docking proteins INS receptor substrates 1 and 2 as mediators of the efficacy of SIT on insulin resistance. DPP-4 activity and GLP-1 levels will be also needed to be measured to confirm that chronic overnutrition elevates plasma DPP-4 activity and DPP-4 inhibitor inhibits plasma DPP-4 activity.

## Figures and Tables

**Table 1:** Baseline and posttreatment parameters of body weight and plasma metabolic markers in control and WD group mice with or without SIT treatment. Values are mean ± SE. ^∗^*P* < 0.05 compared to the CD or CD + SIT group; ^§^*P* < 0.05 compared to the WD group. Sample sizes are noted in parentheses.

Parameter	CD (12)	CD + SIT (12)	WD (12)	WD + SIT (12)
Pretreatment body weight (g)	18.1 ± 0.3	18.2 ± 0.4	18.1 ± 0.3	18.1 ± 0.4
Posttreatment body weight (g)	28.3 ± 1.3	28.8 ± 1.5	51.6 ± 2.5^∗^	44.5 ± 2.6^∗^^,§^
Hepatic weight (mg)	826 ± 32	819 ± 39	2453 ± 196^∗^	2232 ± 188^∗^
Hepatic triglycerides (nmol/g)	15 ± 3	16 ± 3	45 ± 4^∗^	32 ± 4^∗^^,§^
Plasma cholesterol (mg/dL)	82 ± 5	80 ± 6	157 ± 6^∗^	132 ± 8^∗^
Plasma triglycerides (mg/dL)	133 ± 10	137 ± 11	325 ± 16^∗^	269 ± 18^∗^^,§^
Plasma alanine aminotransferase (U/L)	25 ± 2	27 ± 3	82 ± 6^∗^	63 ± 5^∗^^,§^

## Data Availability

The data used to support the findings of this study are available from the corresponding author upon request.
